# Detection capacity of small intestine bacterial or methanogen overgrowth by lactose and fructose breath testing in the adult population

**DOI:** 10.1515/almed-2024-0115

**Published:** 2024-08-09

**Authors:** Emilio José Laserna Mendieta, Verónica Martín Dominguez, Irene Pérez Lucendo, Inmaculada Granero Cremades, Raquel Ferreirós Martínez, Tomás Álvarez Malé, María Ángeles Sanz De Benito, Cecilio Santander

**Affiliations:** Gastroenterology Research Unit, Hospital General de Tomelloso, Tomelloso, Ciudad Real, Spain; Service of Clinical Laboratory, Hospital Universitario La Princesa, Madrid, Spain; Instituto de Investigación Sanitaria La Princesa, Madrid, Spain; Instituto de Investigación Sanitaria de Castilla-La Mancha (IDISCAM), Toledo, Spain; Centro de Investigación Biomédica en Red de Enfermedades Hepáticas y Digestivas (CIBERehd), Madrid, Spain; Department of Gastroenterology, Hospital Universitario La Princesa, Madrid, Spain; Department of Medicine, Universidad Autónoma de Madrid, Madrid, Spain

**Keywords:** fructose malabsorption/intolerance, lactose malabsorption/intolerance, small intestine bacterial overgrowth, intestinal methanogenic overgrowth, exhaled breath test

## Abstract

**Objectives:**

Exhaled breath tests (BTs) are the main diagnostic method for fructose and lactose malabsorption/intolerance (FI and LI, respectively) and for detecting small intestine bacterial or methanogen overgrowth (SIBO/IMO). Although FI/LI-BTs may provide evidence of the presence of SIBO/IMO, there is limited literature evaluating their reliability for this purpose. The objective of this study was to assess the sensitivity and specificity of FI/LI-BTs in detecting SIBO and their concordance with SIBO-BTs in the identification of IMO.

**Methods:**

In this retrospective observational study, FI/LI-BTs and SIBO-BTs performed in the same patients within a period of 6 weeks were selected from 652 gas chromatography-based BTs.

**Results:**

A total of 146 BTs from 67 eligible adult patients were identified. LI-BTs had higher specificity than FI-BT in detecting SIBO (93.8 % vs. 72.7 %). In contrast, FI-BTs showed higher sensitivity (60.0 % vs. 28.6 %) as FI was more frequently established in SIBO-positive patients (70 % vs. 29 %). With regard to IMO, concordance with LI-BT was 100 %, with a 27 % of false negatives on FI-BTs.

**Conclusions:**

Findings suggestive of SIBO or IMO on LI-BTs were highly consistent with those of SIBO-BTs. In contrast, the rate of false positives for SIBO and the rate of false negative for IMO on FI-BTs was 27 % in both cases.

## Introduction

Small intestine bacterial overgrowth (SIBO) is a functional bowel disorder characterized by excessive proliferation of bacterial microbiome in the small intestine. Manifestations of this entity include bloating, flatulence, abdominal pain and diarrhea or constipation [1, 2]. These symptoms can be due to abnormal intestinal permeability and motility, a higher level of inflammation or immune activation, malabsorption of nutrients or fermentation of substrates in the small bowel [3].

In some cases, this overgrowth may be caused by intestinal methanogen overgrowth (IMO) [4]. These methanogenic organisms, primarily *Methanobrevibacter smithii* [5] are not bacteria but archaea. Hence a different term has been suggested to refer to their overgrowth [1]. IMO has been associated with constipation, due to the inhibitory effect of methane (CH_4_) on intestinal peristalsis [6]. A large retrospective study demonstrated that IMO is a different entity from SIBO and that excessive CH_4_ production is more frequent in elderly patients. The study also revealed that malabsorption of nutrients such as vitamin B12 is less frequent in IMO [7]. Additionally, a different antibiotic treatment is indicated for IMO. The combination of neomycin and rifaximin has been proven to be more effective in IMO patients than rifaximin alone, the treatment indicated for SIBO [8, 9].

The gold standard diagnostic study for SIBO is jejunal aspirate culture. However, its use is challenging, as it is an expensive invasive test and a standard cut-off of colony-forming units per ml has not yet been established [3]. Hence, the most widely used study for testing suspected SIBO or IMO is exhaled breath testing (BT). BT is a low priced, non-invasive, easy-to-use test that is widely available in a large number of laboratories. This test measures hydrogen (H_2_), methane (CH_4_), and carbon dioxide (CO_2_), with the latter being used as a correction factor and to verify correct sample collection.

A variety of guidelines and consensus documents are available for the correct performance of BTs, including preparation instructions for patients undergoing a BT. The most relevant guidelines are the American [10] and the European [11]. However, these guidelines use different cut off points for the interpretation of SIBO-BT. Whereas the American guideline establish a rise in H2 of ≥20 parts per million (ppm) from baseline at 90 min, the European guideline only establish early elevation of H_2_≥10–12 ppm from baseline, without providing any time interval. The American guideline further suggest that CH_4_ elevation (≥10 ppm) indicates the presence of IMO, whereas this aspect is not mentioned in the European guideline. The most widely recommended substrate is 50–75 g of glucose or 10–20 g of lactulose. Of note, another substrate similar to lactulose is used in Spain, lactitol. These substrates are widely used for diagnosis of SIBO, as they both are nonabsorbable sugars. A recent meta-analysis revealed a slightly higher diagnostic performance for glucose, as compared to lactulose [12].

As any other test, BTs also have some limitations such as it being an indirect measurement method. In addition, accelerated/reduced orocecal transit (due to physiological factors, previous surgery, use of drugs, and/or diseases) can interfere with results. Other limitations include its lack of correlation with the results of small bowel aspirate culture [13]. In addition, since methanogenic archaea use H_2_ as a substrate for CH_4_ production, the levels of H_2_ are usually low in the presence of IMO; therefore, it is critical that the instruments used to measure exhaled breath gases in BTs also measure CH_4_ concentration [14].

On another note, malabsorption/intolerance to carbohydrates such as fructose (FI) or lactose (LI) can be related to the presence of SIBO and mimic its symptoms, as SIBO can reduce their absorption due to abnormal villi function and/or destruction of intestinal mucosal enzymes [15, 16]. Thus, FI/LI-BTs are frequently requested for patients with suspected SIBO/IMO. FI/LI-BTs may provide evidence suggestive of SIBO or IMO in the form of early H_2_ elevation or high CH_4_ concentrations [17].

Although the American consensus guideline recommend SIBO-BTs to be performed prior to carbohydrate malabsorption/intolerance BTs [10], for practical reasons, and due to the non specificity of symptoms, several BTs are often requested in the same medical consultation. Understanding the concordance between FI/LI-BTs and SIBO-BTs would be useful to avoid repeated BTs, recurrent consultations and delayed diagnosis, thereby reducing costs. However, little attention has been paid to this issue, with most studies having focused on positive cases for FI/LI that could be secondary to SIBO [18, 19].

The objective of this study was to examine whether findings suggestive of SIBO and IMO on FI/LI-BTs in the adult population were consistent with the results of SIBO-BTs. This study will extend knowledge on the sensitivity and specificity of FI/LI-BTs in detecting SIBO and their concordance in identifying IMO.

## Materials and methods

### Patients

A retrospective observational study was performed to analyze the results of the BTs carried out in routine practice at Hospital Universitario La Princesa (HULP) between February 2020 and April 2023 (n=652). The study sample was composed of BTs administered to the same patients within a maximum period of 6 weeks (42 days), with no changes of treatment between the different BTs. The minimum period between BTs was 1 week, as specified in the instructions provided to patients undergoing more than one BT. All BTs were performed in adults older than 16 years.

Data included sex, age at first BT, type of BT performed, time interval (in days) between the first and the last BT, and result of the BT. BTs were excluded from final analysis when results were interpreted by the specialist to be due to poor preparation for BT (upon observation of high baseline values of H_2_ and/or CH_4_ followed by low values in subsequent time points) [20], or when results were associated with incorrect sample collection (levels of CO_2_<2 %).

Patient data was anonymized and extracted solely from the Laboratory Information System. In compliance with current laws and regulations and with the Declaration of Helsinki, clinical records were not inspected.

### Breath testing method

BTs were supplied by Isomed Pharma SL (Madrid, Spain) and involved the collection of a baseline sample of exhaled breath and the ingestion of the following substrates: 25 g of fructose (for FI), 25 g of lactose (for LI) and 10 g of lactitol (for SIBO). Then, a sample of exhaled breath was collected at intervals of 25 min for 175 min (8 samples in total). The study sample included both BTs performed by patients at home (following written instructions provided by a healthcare professional) and BTs carried out in the hospital with monitoring by nursing staff.

Levels of H_2_, CH_4_ and CO_2_ in the exhaled air tubes were measured on a BreathTracker SC gas chromatograph (QuinTron, Milwaukee, WI, USA) available at HULP. The measurement range was 2–150 ppm for H_2_; 2–75 ppm for CH_4_; and 0.1–7.0 % for CO_2_. A value of CO_2_ of 5.5 % was used as a correction factor for H_2_ and CH_4_ concentrations, which were calculated automatically by the analyzer. A gas with a known concentration supplied by Isomed Pharma SL was used for calibration and quality controls of the chromatograph.

### Interpretation of BTs and analysis of concordance

BT results were interpreted in accordance with clinical guidelines, with some modifications [10, 11]. A FI/LI-BT was considered to be suggestive of SIBO when an increase of ≥20 ppm of H_2_ from baseline was observed at 90 min (in our case, at 75 min, which corresponds to the fourth sample collection). Results were suggestive of IMO when CH_4_ values were ≥10 ppm (in our case, when this condition was fulfilled for all time points). The same criteria were applied to identify a positive result for SIBO and IMO on a SIBO-BT. When IMO was identified, SIBO-BTs were not included in the analysis of concordance for the detection of SIBO. The reason is the influence that CH_4_ has in H_2_ concentration, as it is the substrate used to produce CH_4_, and hence, flat curves of H_2_ were obtained in most cases of IMO [4]. In parallel, FI/LI-BTs were considered positive for FI/LI when an increase of ≥20 ppm of H_2_ or ≥10 ppm of CH_4_ was observed with respect to baseline in at least one point of the curve, following the recommendations of the two clinical guidelines.

When a test was positive both on SIBO-BT and FI/LI-BTs, then carbohydrate malabsorption/intolerance could be secondary to the presence of SIBO. The percentage of these cases with respect to the total number of positive results for SIBO was calculated. Malabsorption was separated from intolerance using the validated scale developed by Casellas et al., which includes five symptoms (diarrhea, abdominal cramping, vomiting, audible bowel sounds and flatulence or gas) rated on a 10-point scale according to their intensity. A total score of ≥7 points indicates intolerance, whereas a lower total score indicates malabsorption [21].

To assess concordance between FI/LI-BT and SIBO-BT in detecting SIBO, contingency tables were constructed to calculate the sensitivity, specificity, positive predictive value (PPV), and negative predictive value (NPV) with their 95 % confidence intervals (CI) using the GraphPad Prism version 5.0 (GraphPad Software, San Diego, CA, EEUU). The percentage of coincidence was calculated to assess the level of concordance between FI/LI-BT and SIBO-BT in detecting IMO.

## Results

### Demographic data and characteristics of BTs

Of the 652 BTs performed at our hospital during the study period, 146 were eligible and corresponded to 67 SIBO-BTs, 47 FI-BTs, and 32 LI-BTs. These BTs were performed in 67 patients, of whom 82 % were female, with a mean age of 47.8 ± 16.9 years (range 17.7–85.6).

**Figure 1:**
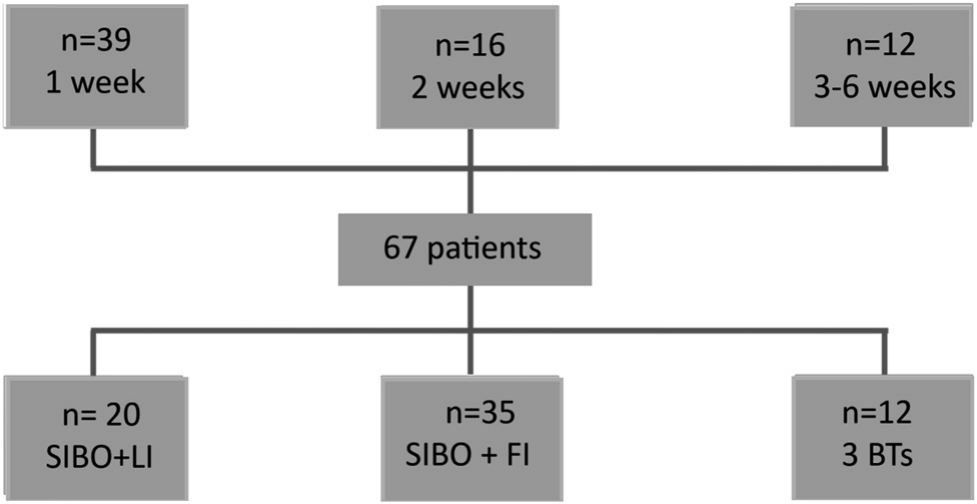
Characteristics of exhaled breath tests (BTs) included in the final analysis according to the type of BT: fructose intolerance (FI); lactose intolerance (LI); or small intestine bacterial overgrowth (SIBO), and time period between the first and the last BT performed in the same patient.

Figure 1 details the types of BTs performed and the time intervals between tests. The majority of patients (52 %) underwent a FI-BT and a SIBO-BT, whereas only 18 % underwent the three types of BTs. The most frequent time interval between BTs was 1 week (58 %), followed by 2 weeks (24 %) and 3–6 weeks (18 %).

In total, 52.9 % of patients with a positive FI-BT also showed intolerance, with this percentage rising to 77.8 % in patients with a positive LI-BT.

### Concordance between FI/LI-BTs and SIBO-BT in detecting SIBO

To evaluate the capacity of FI/LI-BTs to detect SIBO, a comparative study was performed from the results of 32 FI-BTs and 23 LI-BTs.

The sensitivity of FI-BTs was 60.0 % (CI: 26.2–87.8 %) with a specificity of 72.7 % (CI: 49.8–89.3 %), a PPV of 50.0 % (CI: 21.1–78.9 %) and a NPV of 80.0 % (CI: 56.3–94.3 %) (Table 1). Hence, SIBO-BT was negative in 27.3 % of FI-BTs suggestive of SIBO. Seven of the 10 patients with SIBO also had a positive result for FI.

**Table 1:** Contingency table displaying the results obtained for patients who underwent exhaled breath tests for fructose intolerance (FI-BT) and bacterial overgrowth (SIBO-BT).

	Positive SIBO-BT	Negative SIBO-BT	Total
FI-BT suggestive of SIBO	6	6	12
FI-BT not suggestive of SIBO	4	16	20
Total	10	22	32

The number of false positives was lower (6.2 %) in LI-BTs, which indicates a higher specificity. The results of this comparative study are shown in Table 2, reporting a sensitivity of 28.6 % (CI: 3.7–71.0 %), a specificity of 93.8 % (CI: 69.8–99.8 %), a PPV of 66.7 % (CI: 9.4–99.2 %) and a NPV of 75.0 % (CI: 50.9–91.3 %). Of the seven patients with SIBO, only two had a positive LI-BT.

**Table 2:** Contingency table displaying the results obtained for patients who underwent exhaled breath tests for lactose intolerance (LI-BT) and bacterial overgrowth (SIBO-BT).

	Positive SIBO-BT	Negative SIBO-BT	Total
LI-BT suggestive of SIBO	2	1	3
LI-BT not suggestive of SIBO	5	15	20
Total	7	16	23

A summary of sensitivity, specificity, PPV and NPV values for the detection of SIBO in FI/LI-BTs is provided in Table 3.

**Table 3:** Summary of sensitivity, specificity, positive predictive value and negative predictive value for the detection of small intestine bacterial overgrowth by exhaled breath testing (BT) for fructose and lactose intolerance. Values between brackets represent 95 % confidence intervals.

	Fructose BT	Lactose BT
Sensitivity	60.0 % (26.2–87.8 %)	28.6 % (3.7–71.0 %)
Specificity	72.7 % (49.8–89.3 %)	93.8 % (69.8–99.8 %)
Positive predictive value	50.0 % (21.1–78.9 %)	66.7 % (9.4–99.2 %)
Negative predictive value	80.0 % (56.3–94.3 %)	75.0 % (50.9–91.3 %)

Of the patients included for these analyses, only eight underwent the three tests. In six of these patients, the level of concordance among the three tests was 100 %. In two patients, H_2_ elevation on the FI-BTs was detected at 50 min, which suggested the presence of SIBO. In contrast, the SIBO-BT was negative, which was in agreement with the results of LI-BTs.

### Concordance between FI/LI-BTs and SIBO-BTs in detecting IMO

In 20 patients, the SIBO-BT suggested the presence of IMO. A total of 15 and 9 FI-BTs and LI-BTs were available for comparison, respectively, as four of these patients underwent both FI-BT and LI-BT. SIBO-BT was positive for IMO in all cases with a FI/LI-BT suggestive of IMO (Table 4).

**Table 4:** Concordance between the results obtained in the exhaled breath test for fructose (FI**-**BT) and lactose (LI**-**BT) intolerance in patients who underwent a breath test for bacterial overgrowth (SIBO-BT) for evaluating intestinal methanogenic overgrowth (IMO).

	Positive SIBO-BT for IMO	Negative SIBO-BT for IMO	Concordance
FI-BT suggestive of IMO	11	0	43/47 (91,5 %)
FI-BT not suggestive of IMO	4	32	
LI-BT suggestive of IMO	9	0	32/32 (100 %)
LI-BT not suggestive of IMO	0	23	

The level of concordance for the evaluation of IMO was 100 % for LI-BT and 91.5 % for FI-BT, as the latter did not detect 4 cases of IMO, thereby yielding a rate of false negatives of 27 %. In the four patients who underwent the two tests, both FI-BTs and LI-BTs detected the IMO.

## Discussion

BTs are a useful tool to evaluate functional disorders in patients with non-specific gastrointestinal symptoms such as bloating, abdominal pain or flatulence, due to their low cost and simplicity [11]. A study in 1,230 patients in whom underlying gastrointestinal disease had been excluded endoscopically and radiologically, revealed that 45 % had SIBO, FI and/or LI, which were detected by BTs [22].

This study contributes relevant evidence to the limited literature available on findings suggestive of SIBO on FI/LI-BTs. Additionally, this is the first study to provide data on the concordance of these tests in detecting the presence of IMO. The rate of false positives for SIBO on FI-BTs was 27 %. False positives were defined as early H_2_ elevations suggestive of SIBO, which were not confirmed by SIBO-BT with lactitol. In contrast, the rate of false positives on LI-BTs was as low as 6 %, which indicated a high specificity. LI-BT also provided optimal results for the detection of IMO, with a level of concordance of 100 %. In contrast, the rate of false negatives for FI-BTs was 27 %.

IMO can be currently identified with gas chromatographs with capacity to measure both CH_4_ and H_2_. The prevalence of IMO could be 30–60 % of all overgrowths when tested by BT [23]. In our center, IMO accounted for 57 % of all overgrowths detected using this method [24]. According to our experience, when lactitol is used as a substrate, IMO manifests as a plateau where CH_4_ is elevated at baseline, with values ≥10 ppm at all points of the BT curve. In contrast, the American guideline, which are based on lactulose or glucose, defined a positive test for IMO as CH_4_ ≥10 ppm at any point of the curve [10]. On the other hand, the European guideline did not include any recommendation for the detection of IMO [11]. With lactitol, CH_4_ rises in parallel to H_2_. As a result, CH_4_ is frequently ≥10 ppm at 100 min or later. Our interpretation is consistent with that of a recent study that revealed a high sensitivity (86 %) and specificity (100 %) in detecting IMO in patients with baseline samples containing CH_4_≥10 ppm, as measured by glucose and lactulose SIBO-BT [25].

This is not only the first study to evaluate the concordance between FI-/LI-BTs and SIBO-BTs in detecting IMO. In addition, to the best of our knowledge, this is the first study to evaluate in an accurate and precise way the performance of FI-/LI-BTs in detecting SIBO. A previous study already identified SIBO as a factor that increased the probability of LI in patients with chronic diarrhea, although a thorough comparative study was not performed [26]. Previous studies only revealed a higher percentage of positive results for LI or sorbitol intolerance – but not for FI – when patients were found to be positive on SIBO-BT [18]. Evidence has also been provided that SIBO was a frequent condition (75 %) causing LI solely detected by BTs but not by oral lactose tolerance test [19]. According to an Asian study, the prevalence of LI secondary to SIBO was 15 % [27], a slightly lower percentage to that observed in our study (29 %). Finally, a recent study reported a higher percentage of cases of SIBO (28 % vs. 7 % in controls) in patients with lactase deficiency confirmed by biopsy [28]. Our study provides novel data on FI suspected to be secondary to SIBO, with a prevalence of 70 %, or the lower rate of false positives (6 %) between LI-BTs and SIBO-BTs in relation to SIBO, as compared to FI-BTs (27 %).

The main strengths of this study are that it is based on BTs performed over a 3-year period using the same gas chromatograph and in patients in whom different BTs were carried out in a short period of time. A limitation of this study is that H_2_ elevation may be secondary to an accelerated intestinal transit. However, this disorder would affect SIBO-BT and FI/LI-BTs results equally. Additionally, the use of lactitol and exhaled breath sampling every 25 min for the SIBO-BT (which hinders measuring gases at 90 min) do not fully align with the recommendations of the American or European guidelines [10, 11]. As it occurs in most centers, duodenal aspirate cultures were not performed in routine practice; therefore, SIBO-BT results could not be compared against those of this gold standard test. The results reported are applicable to comparative studies of FI/LI-BTs and SIBO-BTs when lactitol is used as substrate. Studies with a larger number of patients and using other substrates (glucose or lactulose) are needed to confirm our findings. The production of hydrogen sulfide was not measured either, and it could be the cause of the presence of flat curves without H_2_ or CH_4_ elevation in five patients, which were interpreted as negative for SIBO and IMO.

In conclusion, this study demonstrates that detection of SIBO or IMO in LI-BT has a high level of concordance with SIBO-BT results. This means that SIBO-BT would not be necessary, which would ultimately prevent delayed diagnosis and treatment. In contrast, FI-BT showed a lower level of concordance with SIBO-BT, as 27 % of cases of detected SIBO will be false positives, and IMO would not be identified in another 27 %. These results highlight the need for highly-trained professionals in the optimal interpretation of BTs. This added to an adequate indication would ensure correct diagnosis and effective treatment, jointly with improved cost-effectiveness of these tests.
